# The Anomalous Mei-yu Rainfall of Summer 2020 from a Circulation Clustering Perspective: Current and Possible Future Prevalence

**DOI:** 10.1007/s00376-021-1086-y

**Published:** 2021-08-31

**Authors:** Robin T. Clark, Peili Wu, Lixia Zhang, Chaofan Li

**Affiliations:** 1 Met Office Hadley Centre, FitzRoy Road, Exeter, EX1 3PB UK; 2 LASG, Institute of Atmospheric Physics (IAP), Chinese Academy of Sciences, Beijing, 100029 China; 3 Collaborative Innovation Center on Forecast and Evaluation of Meteorological Disasters, Nanjing University of Information Science & Technology, Nanjing, 210044 China; 4 Center for Monsoon System Research, Institute of Atmospheric Physics, Chinese Academy of Sciences, Beijing, 10029 China

**Keywords:** circulation clustering, mei-yu front, 2020 summer rainfall, climate projections, 环流分型, 梅雨锋, 2020 夏季降水, 气候预估

## Abstract

Highly unusual amounts of rainfall were seen in the 2020 summer in many parts of China, Japan, and South Korea. At the intercontinental scale, case studies have attributed this exceptional event to a displacement of the climatological western North Pacific subtropical anticyclone, potentially associated Indian Ocean sea surface temperature patterns and a mid-latitude wave train emanating from the North Atlantic. Using clusters of spatial patterns of sea level pressure, we show that an unprecedented 80% of the 2020 summer days in East Asia were dominated by clusters of surface pressure greater than normal over the South China Sea. By examining the rainfall and water vapor fluxes in other years when these clusters were also prevalent, we find that the frequency of these types of clusters was likely to have been largely responsible for the unusual rainfall of 2020. From two ensembles of future climate projections, we show that summers like 2020 in East Asia may become more frequent and considerably wetter in a warmer world with an enhanced moisture supply.

**Electronic supplementary material:**

Supplementary material is available in the online version of this article at 10.1007/s00376-021-1086-y.

## 摘要

2020 年夏季, 中国、 日本和韩国的诸多地区均出现了创纪录的极端强降水. 该事件的个例研究将这一特殊事件归因于西北太平洋副热带反气旋的位置移动, 可能与印度洋海表面温度异常和自北大西洋发出的中纬度波列有关. 本工作通过对东亚逐日海平面气压 (SLP) 空间场的聚类分析, 发现 2020 年夏季南海地区为正 SLP 异常的天气型出现天数达到整个夏季的 80%, 为 1979 年以来之最. 通过与其他环流型所对应的降水和水汽通量异常对比, 发现这类天气型的频繁出现很可能是造成 2020 年梅雨期降水偏多的主要原因. 该研究进一步分析了两个大样本集合气候模拟预估结果, 发现未来在全球进一步增暖、 水分供应相应增加的情景下, 像 2020 年这样的东亚夏季环流可能会出现得更加频繁, 中国降水也会更强.

**Article Highlights:**

- Summer 2020 was dominated to an unprecedented extent, by clusters of days of surface pressure greater than normal in the South China Sea.
- The prevalence of positive surface pressure anomalies over the South China Sea is a potentially useful proxy of such events in the future.
- According to model projections, summers of circulation clusters in China, similar to those of 2020 may become more frequent in the future.
- Projections also suggest potentially large rainfall increases in China in future years when circulation is similar to that of 2020.

## 1. Introduction

Summer 2020 will be remembered across a great part of Asia for its highly unusual rainfall anomalies. A westward extension of the climatological West Pacific Subtropical High pressure (WPSH) persisted throughout the summer (Liu et al., 2020), supporting positive pressure anomalies at the surface (see Fig. 1) over the South China Sea, Indochina, and the Bay of Bengal. At this latitude, the rainfall was less than normal. To the west, over southern India, and to the north, between 27°N and 35°N however, it was wetter than normal over an unusually elongated region stretching 5000 km from the Himalayas, through to Japan (Fig. 1a) including the Yangtze river basin, which had its wettest summer since at least 1961 (Liu et al., 2020).

※ This paper is a contribution to the special issue on Summer 2020: Record Rainfall in Asia—Mechanisms, Predictability and Impacts.

The rainfall itself was a consequence of the mei-yu front, a west-east zone within which northward moving warm, moist air from the northern subtropics is forced to ascend whenmeeting cooler, denser air from mid and high-latitudes (Zheng et al., 2008). The front evolves each summer, gradually moving north, in step with the East Asian Summer Monsoon (Qian and Lee, 2000; Ding and Chan, 2005). Anomalies in this evolution thus result in anomalies of rainfall over eastern Asia (Zhang et al., 2018), with the behavior of the front during 2020 being an example. In the 2020 event, this behavior appears to have been influenced from both the southern and northern sides of the front. Takaya et al. (2020) provided evidence that a subtropical role in the 2020 event originated from warmer than normal Indian Ocean surface temperatures. Liu et al. (2020) highlighted a mid-latitude role in the form of a wave train of circulation anomalies emanating from a positive phase of the summer NAO (North Atlantic Oscillation, Folland et al., 2009) circulation over Europe.

Events of the type similar to that of the 2020 summer are notorious for their often devastating societal and economic impacts (e.g. Guo et al., 2020). Understanding their meteorological causes and quantifying their potential predictability is thus of paramount importance in order to improve future resilience against them. Direct rainfall predictions remain a challenge, although seasonal prediction models have shown some promising results (e.g. Li et al., 2016; Martin et al., 2020). Alternative approaches, including the use of climate indices and dynamic regimes, may also be beneficial (e.g. Camp et al., 2019; Clark et al., 2021) since climate models are usually more successful at simulating large scale, rather than small scale phenomena. To support these efforts, we focus on the 2020 summer from a circulation clustering perspective to develop our understanding of some additional, unusual aspects of this summer. These include the transport of moisture into China and quantification of the rarity of the responsible circulation regime. The same strategy is also employed to assess the risk of similar events occurring in the future, using two ensembles of climate projections. This exercise is highly warranted since the hydrological cycle and its associated rainfall is expected to intensify in a globally warmed world (Held and Soden, 2006; Wu et al., 2010, 2013).

The remainder of this paper is organized as follows. Section 2 outlines the datasets and methods used in this study, section 3 reveals the results of the analysis, section 4 provides a discussion of the results, and section 5 provides a summary of the research.

## 2. Data and methods

Summer climate in Asia is complex with a great variety of thermodynamic and dynamic processes occurring simultaneously and often interacting with each other. Examining its spatial variability including, for example, how 2020 compares to other years, is thus surprisingly difficult, as evidenced by the great variety of methods used in the literature. Examples include indices, such as the Wang-Fan index (Wang and Fan, 1999) or the East Asian Summer Monsoon Index (Wang et al., 2008) which are often used to quantify monsoon strength. These are usually single numbers, rather than spatial fields and are consequentially often too blunt. Clark et al. (2021) showed, for example, that specific values of the Wang-Fan index can occur during days with a wide variety of circulation patterns. Alternative spatial fields, such as those of the variability of surface pressure, geopotential height, and wind velocity can be difficult to interpret in terms of impacts and are rarely spatially coherent.

To handle these types of issues, we use the approach of clustering as described in Clark et al. (2021) in which daily June, July, and August fields of sea level pressure from the ECMWF ERA5 (Hersbach et al., 2020) reanalysis are combined into eight collections (hereafter "clusters") using the k-means clustering technique. With new data now available from 2019 and 2020, the clusters of Clark et al. (2021) have been updated, using ERA5 output from 1979 onwards. Throughout this article, fields from ERA5 will be used as a representation of the observations of 2020 due to their spatial coverage and corresponding associated fields, for which gridded observations (of moisture fluxes, for example) are not yet readily available.

To examine how summers like 2020 might play out in a future warming world, we use model simulations from two ensembles of fully coupled ocean-atmosphere models. The first (denoted PPE), consists of a collection of 15 perturbed physics variants of the Met Office HadGEM3-GC3.05 model (Yamazaki et al., 2021) driven by observed greenhouse gas concentrations up to 2005 and following the (Relative Concentration Pathway) RCP8.5 (Moss et al., 2008) scenario to 2100. The second ensemble consists of simulations contributed to the CMIP6 (Eyring et al., 2016) archive, following a (Shared Socioeconomic Pathway) SSP5-8.5 (O'Neill et al., 2016) pathway, very similar to that of RCP8.5. Both are representative of a world of future rapid economic development and convergence. CMIP6 models, whose simulations were used for the analysis (based on data available at the time of doing the analysis) are shown in Table 1. Models were divided according to their resolution to gain an insight into whether their resolution had any effect on results. A single realization from each model was used.

## 3. Results

### 3.1. *Circulation and moisture transport anomalies directly affecting China*

Before performing any circulation clustering, we briefly examine some aspects of the 2020 summer, shown in Fig. 1. A westward zonal wind anomaly (compared to its climate, shown in Fig. S1e of the Electronic supplementary material, ESM) at 850 hPa was seen over the Philippines (Fig. 1b), the southern half of the South China Sea and

**Table 1**. CMIP6 models contributing to Fig. 6 and supplementary Figs. S6 and S7.

| | Horizontal resolution | Model names | Total number of models |
|---|---|---|---|
| Sea level pressure (SLP) | Greater than approximately 250 km | CNRM-CM6-1-HR, NorESM2-MM, EC-Earth3, GFDL-CM4, CMCC-CM2-SR5, TaiESM1, MRI-ESM2-0, BCC-CSM2-MR, CESM2-WACCM, CNRM-CM6-1, CNRM-ESM2-1, GFDL-ESM4, CESM2, HadGEM3-GC31-MM, EC-Earth3-Veg | 15 |
| | Approximately 250 km | NorESM2-LM, INM-CM5-0, MPI-ESM1-2-LR, IPSL-CM6A-LR, FGOALS-g3, NESM3, MIROC6, ACCESS-CM2,ACCESS-ESM1-5,UKESM1-0-LL | 10 |
| Precipitation | Greater than approximately 250 km | As for SLP, but without CMCC-CM2-SR5, TaiESM1, CESM2-WACCM, CESM2, HadGEM3-GC31-MM, EC-Earth3-Veg | 9 |
| | Approximately 250 km | As for SLP, but without INM-CM5-0 | 9 |

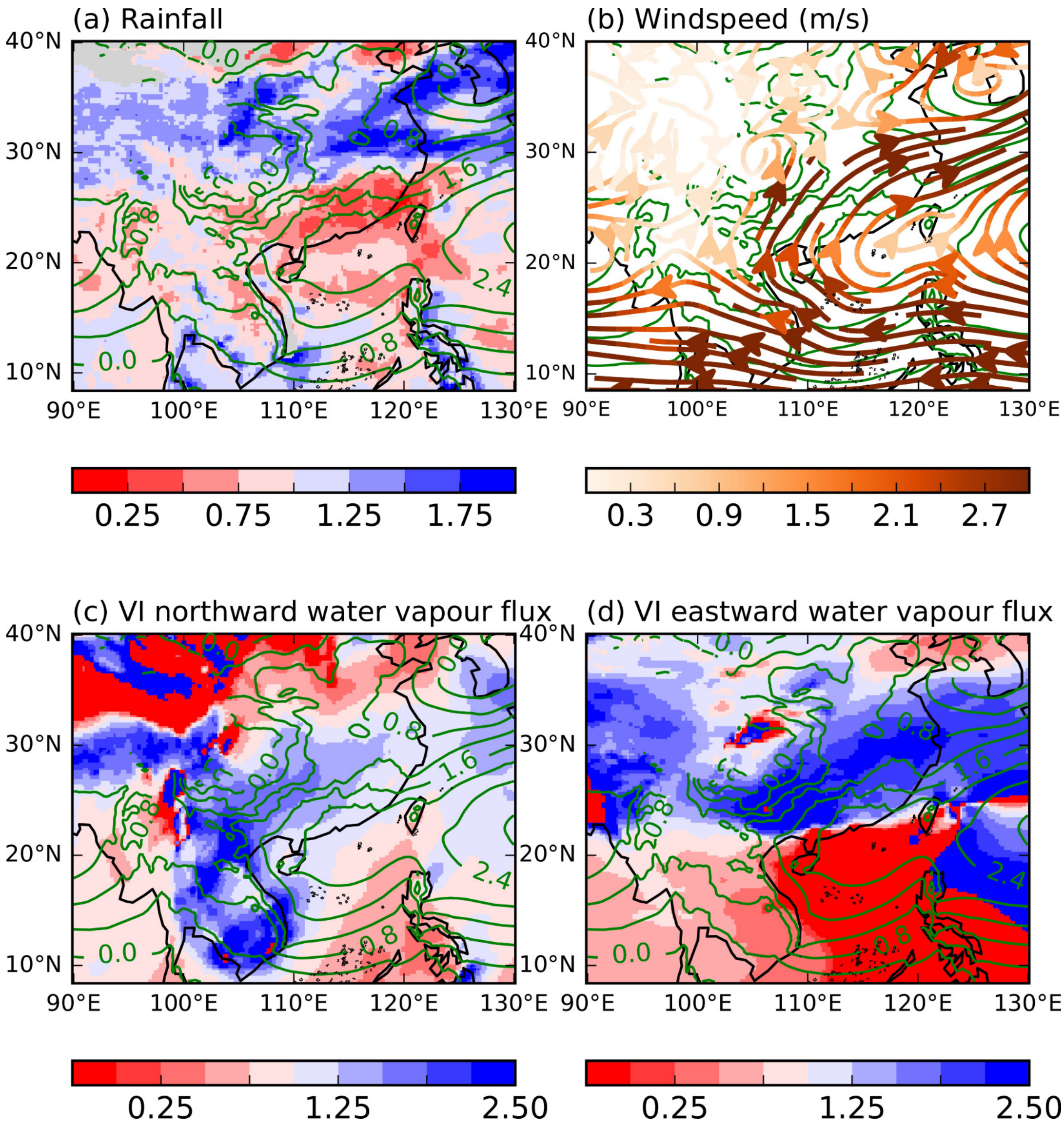

**Fig. 1**. (a) ERA5 rainfall and (c), (d) vertically integrated northward and eastward water vapor flux (color shading) of 15 June to 15 August 2020 as a fraction of the corresponding 1979 to 2020 climatology. (b) Streamlines of 850 hPa wind speed anomalies (from 1979 to 2020 climatology) on the same days as in panels (a), (c), and (d). Green contours show equivalent sea level pressure anomalies (0.4 hPa intervals). Rainfall in (a) is masked (grey) where the climatology is less than 1.0 mm d$^{-1}$. Pressure contours are masked (to avoid noise) in grid boxes with an altitude greater than 3000 m above sea level.

Indochina, with the anomaly streamlines approximately parallel to the pressure anomalies. This wind anomaly matches the westward anomaly of the zonal water vapor flux seen in Fig. 1d, which would have transported moisture westwards from the South China Sea towards Indochina. The westward extent of this can be seen in Fig. S1f in the ESM. The anomalous northward water vapor flux (Fig. 1c) suggests that this anomalous moisture then passed into southern China, in addition to that which climatologically (see Fig. S1c) passes directly from the South China Sea. Over southern China, on the northern side of the surface pressure anomaly over the South China Sea, the eastward zonal wind was greater than usual (Fig. 1b), This eastward wind anomaly appears to have also manifested itself in the eastward water vapor flux (Fig. 1d) which would have resulted in anomalous moisture passing zonally along the mei-yu front, enhancing the rainfall over parts of eastern China under the front.

### 3.2. *Initial cluster analysis of 2020*

To compare the circulation of the 2020 summer to that of other years, we use the clustering approach described in section two. For reference, Fig. 2 shows the eight clusters of daily ERA sea level pressure from all June, July, and August (JJA) days from 1979 to 2020. They are very similar to those generated, in the absence of data, from 2019 and 2020 in Clark et al. (2021). A chart showing how the cluster occurrences have been distributed throughout the period is given in Fig. 3a. A rich variety of circulation types

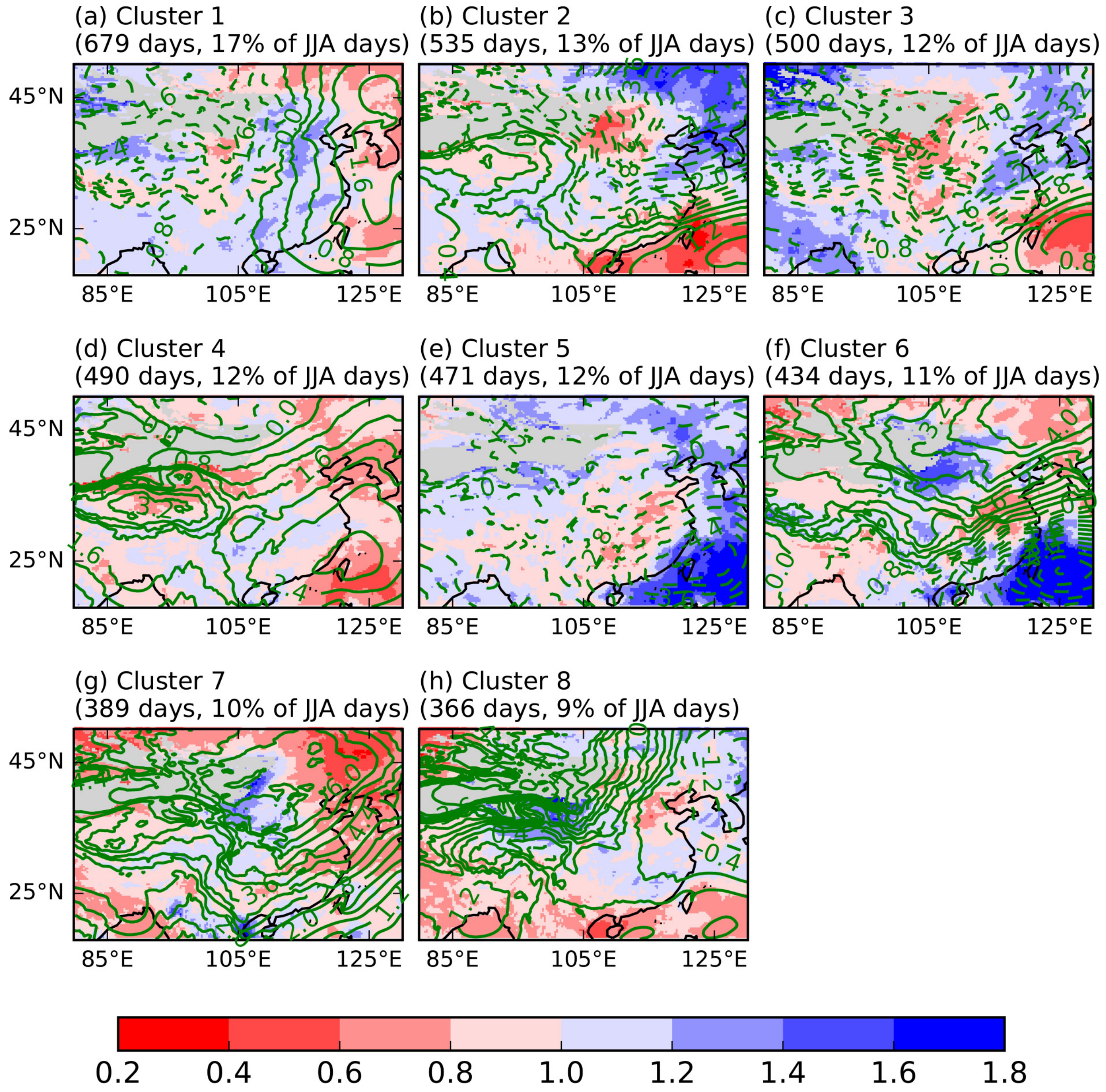

**Fig. 2**. ERA5 rainfall averaged over JJA (1979 to 2020) days in each cluster, as a fraction of overall (regardless of cluster) JJA climate. A value of 2.0, for example, shows rainfall twice the climate. A mask (grey) has been applied where JJA rainfall climate is less than 1.0 mm d$^{-1}$. Dashed and solid contour lines show negative and positive anomalies, respectively, relative to the mean ERA5 sea level surface pressure anomaly (0.4 hPa intervals) in each cluster.

is seen in almost all years, clearly illustrating the complexity of issues mentioned early in section two. There are, however, some patterns. Clusters 1, 2, 3, and 4, for example, typically occur earlier in summer than clusters 5, 6. Clusters 7 and 8 typically occur late in summer. In some years, there are also highly unusual sequences of days when specific clusters persisted much longer than normal. Examples of these include cluster 5 in 1985, and cluster 7 in June 2004 and August 1998, 2010, and 2014. The June 2004 occurrences of cluster 7 appear to have been exceptional, given that this cluster is very rare in June.

Considering the summer of 2020, some details emerge quite easily. Clusters 5 and 6, for example, are almost entirely absent, even during August, when they typically occur. The difference between 2020 and the previous two years (2018 and 2019) in early August is striking. An unusual persistence of cluster 4 in late July 2020 is also shown. Cluster 4 was also more frequent than normal in both June and August. Figure 3b allows the frequency of each cluster in 2020 to be compared with the overall mean (which can be considered as a climatology) and the linearly calculated trend of the 42 year from 1979 to 2020. It shows that clusters 1, 2, and 4 were unusually frequent in 2020; for clusters 2 and 4, but not cluster 1, this matched the sign of the trend of their frequency.

To examine transitions between clusters, Fig. S2 in the ESM gives bar graphs showing the prevalence of clusters of the day immediately following days of those clusters (1 to 4) which were most frequent in 2020. Climatologically, individual days of a cluster are most likely to be immediately followed by a day of the same cluster. This pattern also occurred in 2020. In 2020 however, this occurred even more often than normal for days of clusters 1 and 4 (see Figs. S2a, d in the ESM). For these two clusters, though, the pattern was stronger in other years (2010 and 2005 respectively) when approximately 80% of the days of these two clusters were followed by an additional day of the same cluster. When days of 2020 did transition to a different cluster they tended to evolve almost exclusively into other types characterized by positive surface pressure anomalies over the South China Sea. There are hints that a weak cyclical pattern also occurred in 2020. When days of cluster 1 were not immediately followed by an additional day of cluster 1, they most often evolved into at least a day of either cluster 4 or 2, typically characterized by either strengthening or weakening, respectively, of the positive pressure anomalies over the South China Sea (Figs. 2b, d). Days of cluster 4 subsequently often evolved into those of cluster 2 and days of cluster 2 tended to evolve into days of either clusters 1 or 3. Interestingly, all of those which evolved into

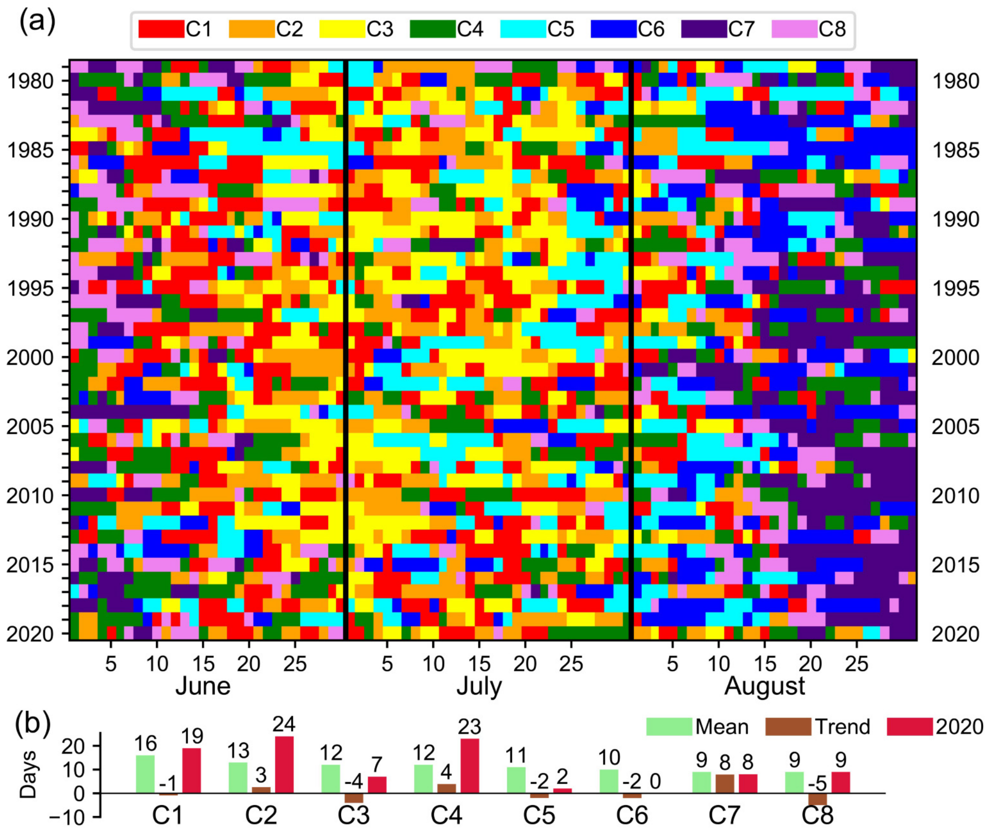

**Fig. 3**. (a) Identities of the ERA5 sea level pressure clusters of Fig. 2 on each June, July, and August day of each 1979 to 2020 year. (b) Statistics (green and brown) of the frequency of each cluster in JJA of each year, and in 2020 JJA (red). Trend values show the linear trend per 10 years, rounded to the nearest whole number.

cluster 3 subsequently evolved into cluster 1.

### 3.3. *Reconstructing 2020 rainfall from cluster rainfall and the role of 850 hPa zonal flow*

In addition to indicating the mean sea level pressure in each cluster, Fig. 2 also provides the mean rainfall in each cluster, expressed as a fraction of the overall mean of all JJA days from the 42-years spanning 1979 to 2020. The spatial patterns of rainfall are clearly related to the magnitude of the pressure anomalies, for example, over the West Pacific between 20°N and 30°N in clusters 2 to 6 (see Figs. 2b–f). Although each cluster is, by definition, a collection of several hundred differing days grouped together solely by the sea level pressure patterns, it is interesting to examine how well the 2020 rainfall shown in Fig. 1a can be reconstructed, using a simple weighted average of the rainfall means of Fig. 2, where the weighting is given according to the frequencies of the clusters in 2020. However, although the reconstructed rainfall has similarities with that of Fig. 1a (see Fig. S3 in the ESM), with a moderate spatial correlation (0.31 and 0.43 depending on whether the rainfall used from each cluster was expressed in absolute terms or as a fraction of the overall climate), there are two very large differences. Firstly, the spatial variability of the reconstructed rainfall is much smaller, resulting from the use of the means of the cluster-dependent rainfall rather than information from the full distribution of days within each cluster. Secondly, the north-south dipole of the wetter and drier than climatology conditions, seen in Fig 1a, is also displaced. It is not surprising that none of the clusters of Fig. 2 show a spatial pattern of rainfall deficit in southeast China as pronounced as seen in Fig. 1a, considering how unusual the 2020 summer was. To further investigate this, Fig. 4 provides maps of the rainfall and 850 hPa zonal wind, during the 2020 summer in each of the four most frequent clusters of 2020, relative to what usually happens in each cluster. In all four, an enhancement of the characteristics of the rainfall in eastern China, shown in Figs. 2a–d, was seen (Fig. 4 left column). Immediately to the north and south of these rainfall enhancements, dipoles of opposing enhancement of the zonal wind were also seen (Fig. 4 right column), suggesting a propensity for additional (i.e. greater than typically expected), local scale cyclonicity. This appeared particularly prevalent in clusters 1 and 3 where meridional gradients of the zonal wind enhancements were remarkably sharp. As explained by Chou et al. (1990), the anomalous westerly wind, immediately to the south of the rainfall, would have favored further ascent and decent anomalies on the north and south sides of the low-level jet, respectively, enhancing the corresponding anomalies of rainfall.

### 3.4. *Grouping of the clusters*

In light of the information from the above sections concerning the roles of clusters 1, 2, 3, and 4 in 2020, we now group the clusters for additional analysis. This can be done statistically using dendrograms applied to the pressure anomalies of the whole domain shown in Fig. 2. However, for examining the 2020 summer an approach focused on eastern China, where the rainfall anomalies were greatest, appeared to be more beneficial. The proximity of the West Pacific subtropical anticyclone to the region impacted is already known to have a large influence on the rainfall in the region (Rodwell and Hoskins, 2001). As highlighted by Lu (2001), Kim et al. (2002), and Preethi et al. (2017), westward extensions of the anticyclone strongly favor greater than normal rainfall over East Asia and the Korean peninsula with moisture transported from the South China Sea and West Pacific. Although primarily a feature at 500 hPa, the variability of the anticyclone also manifests itself through surface pressure anomalies over the South China Sea. For examining the 2020 summer in East Asia, it is thus appropriate to focus on this region for grouping the clusters. In clusters 1, 2, 3, and 4 (denoted Cluster Group CG1234), the surface pressure in the region is greater than normal (see Figs. 2a–d). In clusters 5 and 6 (denoted CG56), the pressure is less than normal (see Figs. 2e, f). The remaining clusters, 7 and 8 (denoted CG78), show no significant anomalies in the region but are characterized by positive anomalies over northwest China. By grouping the clusters in this way, the information of Fig. 3a becomes much clearer, as shown in Fig. 5a, with the 2020 summer dominated by days of the CG1234 cluster group. Of the 62 days, from 15 June to 15 August 2020, 59 of them were representative of this group of clusters and generally corresponded to the time when most of the rainfall occurred in eastern China. Furthermore, the second group (CG56) barely occurred at all in JJA 2020. From Fig. 5b, the cluster group occurrences during 2020, relative to the means, appear unrelated to the trends of the past 42 years.

Supporting the grouping of the clusters, Fig. S4 in the ESM shows anomalies of the 500 hPa geopotential height in the three groups. The position of the climatological WPSH is clearly westwardly displaced and stronger than usual during the days of the CG1234 group. During days of the CG56 group, it is weaker than normal and eastwardly displaced.

An alternative option of simply producing three clusters instead of eight clusters when clustering the daily sea level pressure fields was also attempted. However, this approach produced clusters with little spatial diversity (see Fig. S5 in the ESM). The distinctive negative pressure and associated positive rainfall anomalies over the South China Sea for example, in clusters 5 and 6 of the original 8 clusters, visible in Figs. 2e, f are difficult to identify in Fig. S5. The lack of diversity consequently limits their usefulness in examining extreme events such as those of the 2020 summer in eastern Asia.

### 3.5. *Distributions of cluster group frequencies*

Figures 6a, c, and e give distributions of the frequency of each of the three groups of clusters discussed above for the JJA period of each year. From the green bars of Fig. 6a, for example, it can be seen that during each of the 11 different years, there were between 50 and 55 days of the

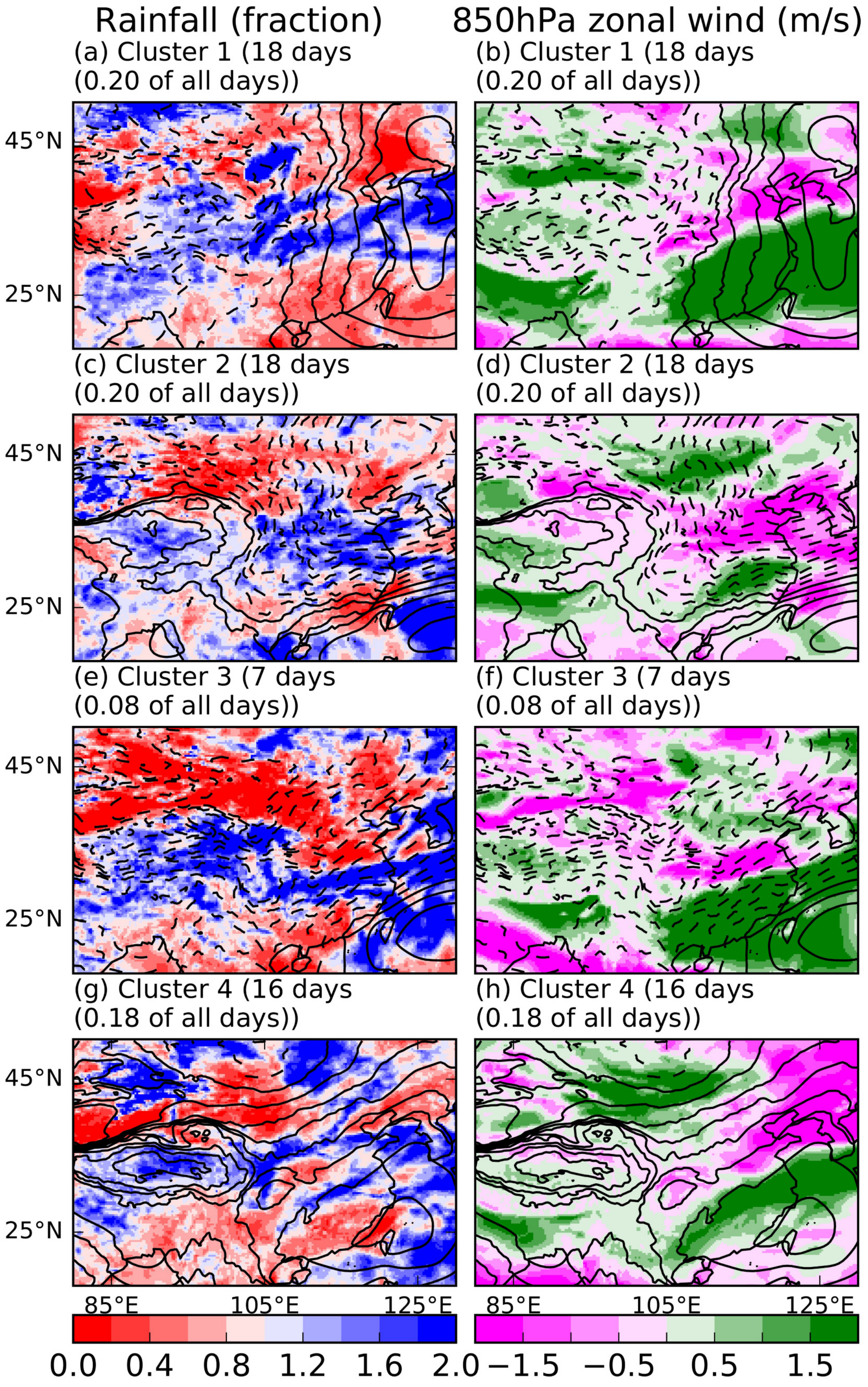

**Fig. 4**. ERA5 rainfall and 850 hPa zonal wind averaged over the days spanning 15 June to 15 August 2020 in each cluster. The rainfall and wind are shown as a fraction and anomaly respectively, of the cluster specific rainfall, compiled from all relevant 1979 to 2020 JJA days.

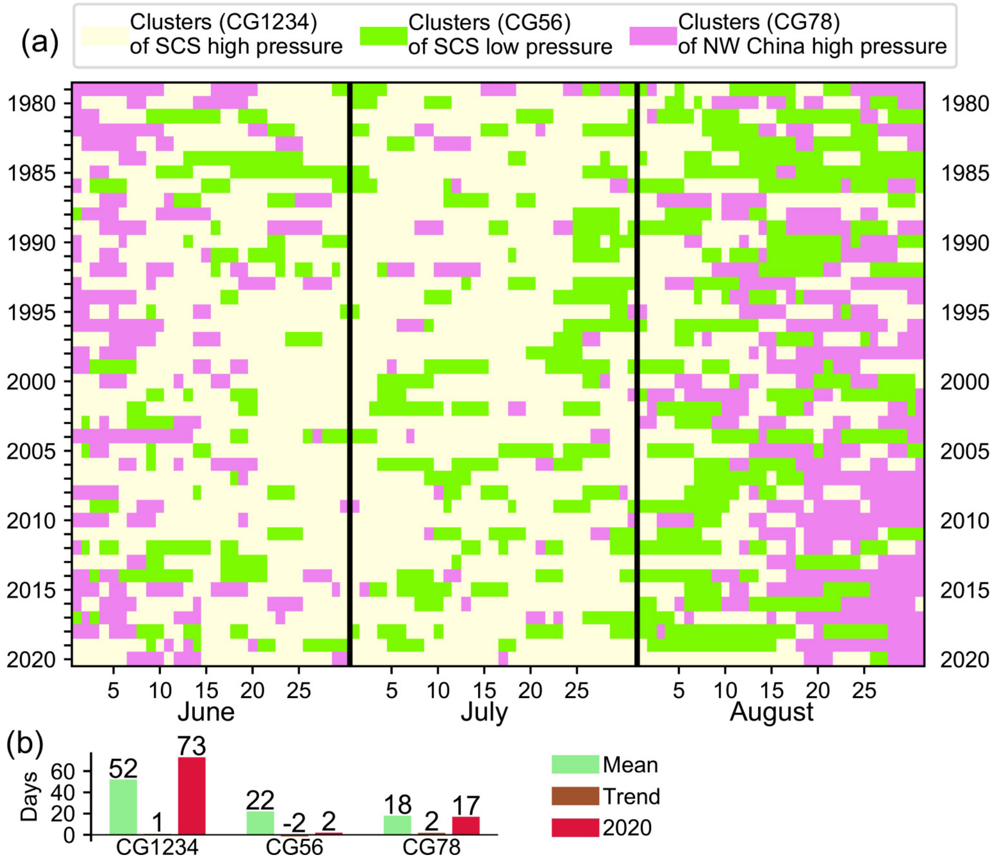

**Fig. 5**. As Fig. 3 but of the clusters of Fig. 2 grouped as follows: CG1234: clusters 1, 2, 3, and 4, CG56: Clusters 5 and 6, CG78: Clusters 7 and 8. SCS: South China Sea.

CG1234 anomaly group according to ERA5, roughly equating to between 54% and 59% of the 92 days in each annual period. Furthermore, in every year at least a third of the 92 days, were of the same group of clusters. In 2020, 73 of the 92 days (or 79%), according to ERA5 (the red dot in Fig. 6a), were of the group. In contrast, only two of the ERA5 JJA days of 2020 (Fig. 6b) were of the group of the two CG56 clusters, a group which typically occurs on 20 to 25 of the 92 days in JJA of each year. The group of clusters of high pressure anomalies over northwest China (CG78) typically occurs outside of the 15 June to 15 August period, both before and after. Therefore, their occurrence appears to have had little relevance during the mid-summer wet period of 2020.

The light blue and pink lines of Figs. 6a, c, and e are the equivalent distributions from the PPE and CMIP6 (using models of all resolutions) ensembles described in section two, for the period 1979–2020. To produce these, each day of each simulation during the JJA period was compared (using Euclidean distance) to the centroids of the original eight clusters (shown in Fig. 2) to determine the most likely cluster of each day. The clusters of these days were then grouped in the same way as was done for those of the ERA5 reanalysis. The PPE and CMIP6 ensembles both appear to reproduce the shape and location of the ERA5 distributions remarkably well, especially when one considers that the 2006 to 2020 part of the simulations is forced according to a future scenario, rather than by observations. Given the observed impacts of the 2020 summer, the future frequency of days of the CG1234 group is of interest. With this in mind, we note that the prevalence of years with larger numbers of days of this group is, according to the PPE, greater for the future 2058 to 2099 time slice. For the CMIP6 models, the future prevalence appears unchanged. Additional plots (not shown), of the CMIP6 models according to their resolution (shown in Table 1) yield very similar results. A caveat here, however, is that only a single realization from each CMIP6 model was used for the analysis. The corresponding future rainfall of this time slice is discussed later.

The right-hand column of Fig. 6 gives the ERA5 rainfall for each of the three groups of clusters as a fraction of the climatological (i.e. regardless of cluster) rainfall . The first (Fig. 6b) has wetter than normal conditions over central and eastern China. The fractions, however, are no greater than 1.2. Much greater fractions are seen (Fig. 6d), for example, in the second group (CG56), over the west Pacific, associated with the cyclonic pressure anomaly. Interestingly, over eastern China, however, rainfall deficits, compared to climate are seen for this second group. In most years, the occurrence of days of this group of clusters would thus be expected to provide respite in eastern China from the rainfall occurring during days of the first (CG1234 anom-

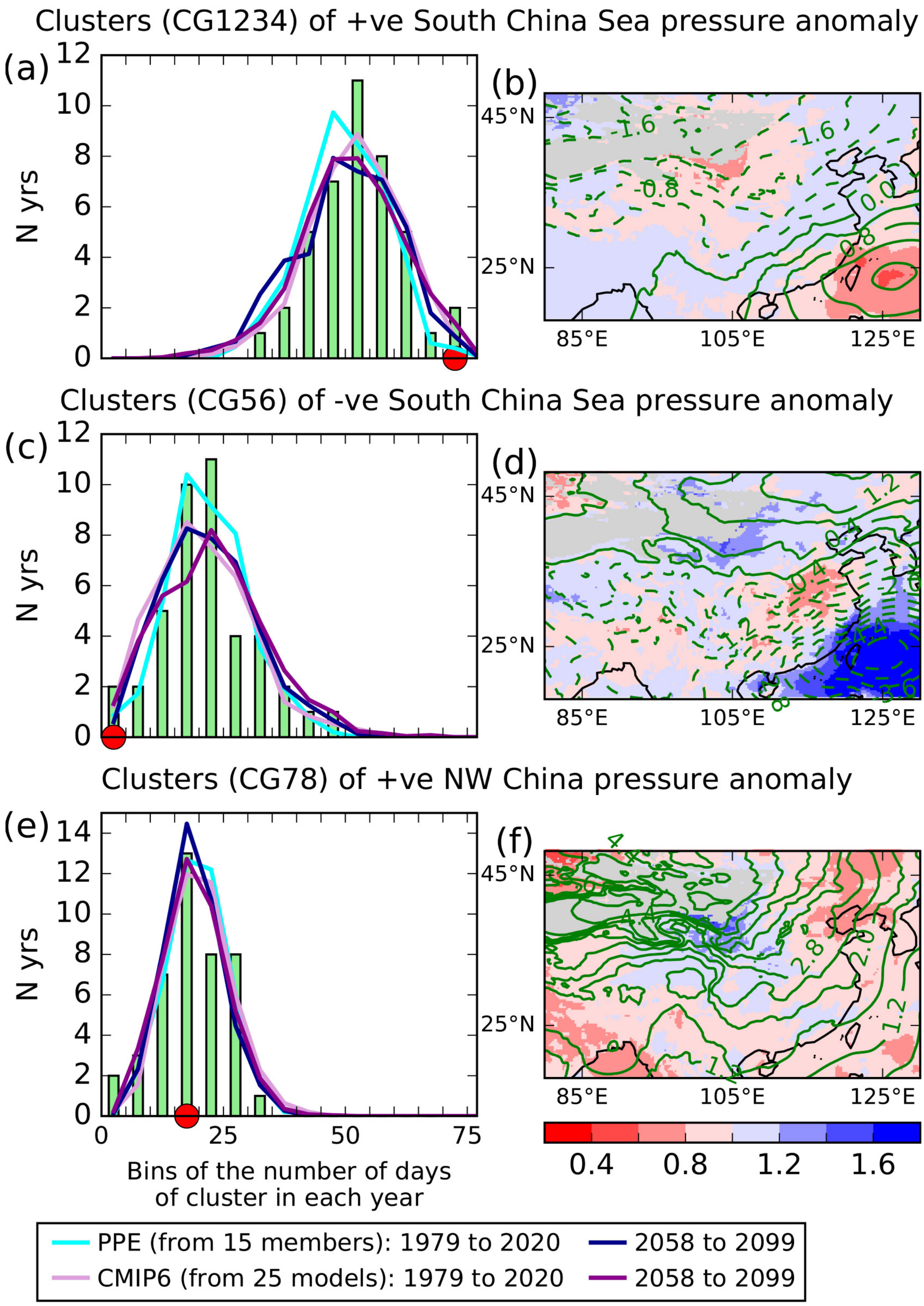

**Fig. 6**. (a), (c), and (e) Distributions of the number of JJA days in individual years, in the three groups of clusters (CG1234, CG56, and CG78), indicated in the row titles. Vertical green bars show ERA5 distribution. The red dot shows the number of days in the ERA5 reanalysis, of each cluster in 2020. PPE (see Yamazaki et al.) lines are of an RCP8.5 Relative Concentration Pathway. CMIP6 lines are of the SSP5-8.5 Social Economic Pathway. PPE and CMIP6 lines shown are ensemble means of distributions (distributions calculated separately for each simulation). (b), (d), and (f) Rainfall in the three cluster groups of (a), (c), and (e) as a fraction of overall climatological rainfall. A mask (grey) has been applied where JJA rainfall climate is less than 1.0 mm d$^{-1}$. Green contours show equivalent sea level pressure anomalies (0.4 hPa intervals).

aly) group. The absence of days in the second group in 2020, especially in July, would have reduced the likelihood of such respites occurring. Mean days of the third group (of CG78 clusters) are slightly drier than normal(Fig. 6f). However, these occurred in early June, before the main rainfall period in the regions most impacted by flooding, and then again in late August, after the rainfall had moved north. Their usefulness as a respite was thus very limited.

## 4. Discussion

### 4.1. *Summers with circulation similar to that of 2020*

We now examine how the prevalence of the CG1234 anomaly group is related to the rainfall and water vapor fluxes of the 2020 summer. We do this by focusing on the five years (1995, 1998, 2003, 2010, and 2013) in which the frequency of this group of clusters was most similar to 2020. The means of these are given in Fig. 7. The precipitation and moisture fluxes are expressed as a fraction (Fig. 7a, c, and e) of the overall climate (regardless of cluster) and are remarkably similar to those of Fig. 1. In particular, the spatial water vapor flux patterns show very clearly how the frequency of the CG1234 group could potentially be used as a proxy for the fluxes. Furthermore, in these five years, the northward and eastward water vapor fluxes over Indochina and southern China, respectively, are even greater than their own cluster-specific climate (see Figs. 7d, f), suggesting that the flux magnitudes are themselves related to the circulation cluster group frequency.

### 4.2. *Future precipitation in years of circulation similar to that of 2020*

In a warmer world, the atmosphere, due to the Clausius-Clapeyron relation, has a greater capacity to hold and transport water vapor from moisture sources to destination regions (Held and Soden, 2006). In the absence of constraints on the moisture sources, rainfall in these destination regions is thus likely to increase. Due to this, and the impacts of the 2020 floods, we now examine how summer rainfall in years with a circulation similar to that of 2020 may change in a future time slice (2058 to 2099). This is useful since we have already seen indications of an increase in the frequency of these types of summers in the future in the PPE ensemble (Fig. 6a).

The top row of Fig. 8 gives the mean rainfall of the five years in the 2058 to 2099 PPE time slice in which the frequency of the CG1234 cluster group is the most frequent. The values are expressed as fractions of the equivalent rainfall from the 1979 to 2020 PPE time slice. The identities of the years were determined independently for each simulation. These five years aretherefore indicative of how the conditions that were observed in 2020 summer could change in the future. Results are given as lower, median, and upper percentile estimates from across the PPE ensemble. Of the three estimates, confidence will naturally be greater in the median. However, the lower and especially the upper estimates should not be ignored because of their potential impacts should they occur. The median estimate (Fig. 8b) strongly suggests that future 2020-circulation-type summers will be wetter than their corresponding recent-past equivalents. From the upper estimate, there is a low (10%) risk of many locations even experiencing 1.5 times as much rainfall as in 2020. Considering that the values in Figs. 8a–c are expressed as fractions relative to the rainfall in years of circulation similar to the extremely wet 2020 summer, suggests that adapting to such increases could potentially be very challenging.

The bottom row of Fig. 8 shows the future rainfall as a fraction of the overall 1979 to 2020 model climatology (calculated independently for each simulation). This allows an approximate visual comparison with Figs. 1a, 6b, and 7a. In almost all regions of China, rainfall is likely to exceed the overall 1979 to 2020 climatology during these years. A resulting expansion of the area impacted by the type of rainfall event which occurred in 2020 thus looks likely, presenting a further additional challenge.

A caveat, however, arises from how the estimates from across the ensemble are calculated. To produce these, the future rainfall fractions (of the 1979 to 2020 period) were ranked separately for each grid box. Spatial coherence between regions is thus not guaranteed. The simulation contributing to the greatest increase in one region, may not be the same elsewhere. Additionally, the PPE ensemble is small, and its range of responses may be skewed as a result of the parameters perturbed. As mentioned in section two, the PPE is also built from a single model. The range of estimates is thus likely to be narrower than that which might be obtained from an ensemble of PPEs built from multiple models. To quantify such estimates is beyond the scope of this study. However, it is possible to get an approximate, initial estimate from the inclusion of a single realization from different models. Figures S6 and S7 in the ESM give the equivalent estimates of Fig. 8, but from the CMIP6 models (see section two for their identities). The increases of the median and upper rainfall estimates are smaller than those from the PPE but their likelihood over eastern and northeastern China appears robust. The CMIP6 model resolution has a small effect (from comparing Figs. S6 and S7) with models of higher resolution having slightly larger increases.

## 5. Conclusions

From mid-June to early August 2020, a large part of East Asia experienced an unusually wet summer as a result of anomalous moisture transported from the South China Sea into southern China in addition to moisture from the Bay of Bengal. The component from the South China Sea appears to have been enhanced by an unusually westward displacement of the climatological West Pacific subtropical anticyclone.

Using clusters of sea level pressure compiled from 42 years of ERA5 re-analyses, the 2020 summer in East Asia,

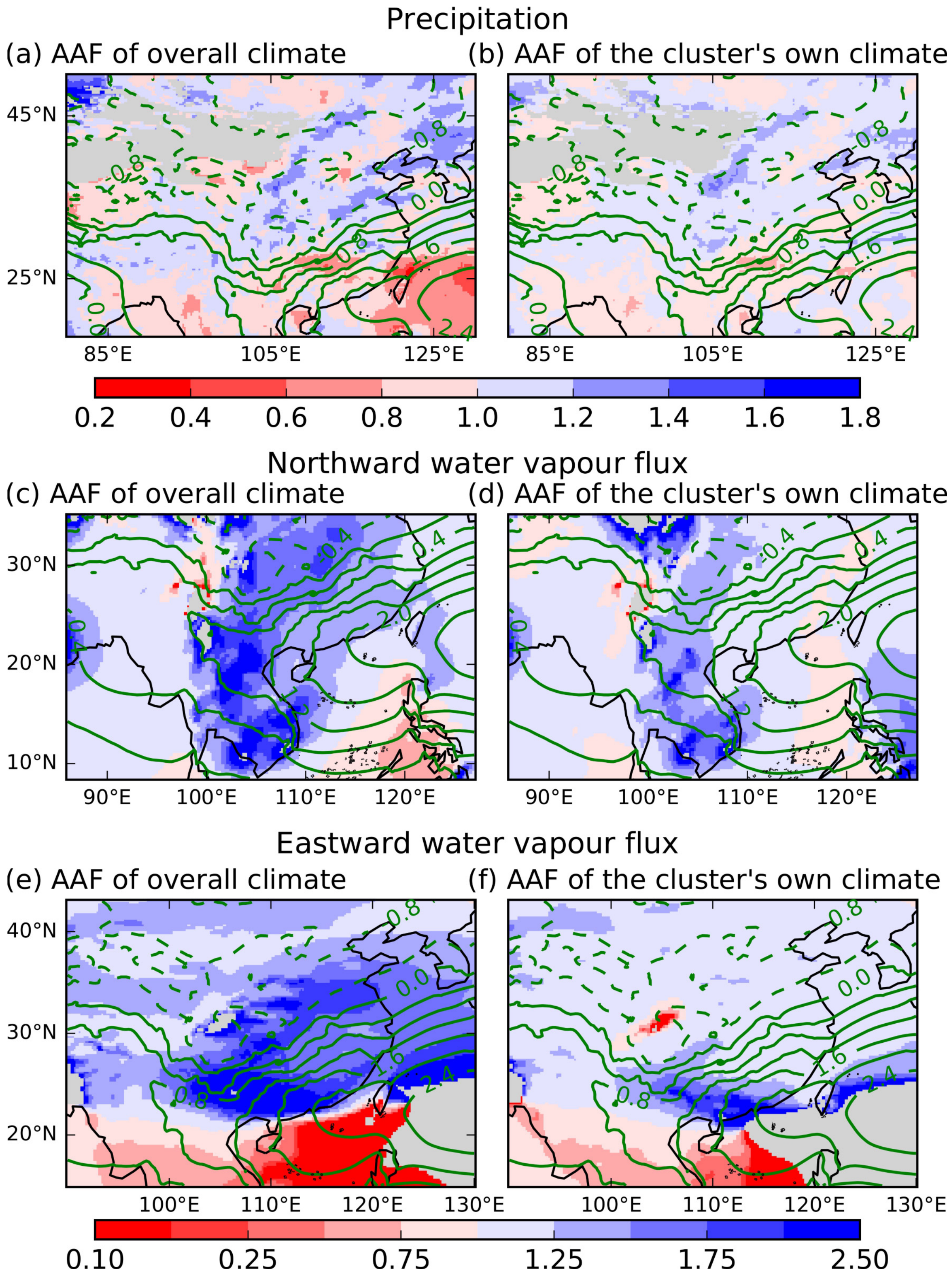

**Fig. 7**. ERA5 precipitation and water vapor fluxes for days spanning 15 June to 15 August for cluster group CG1234 of clusters of surface pressure greater than normal over the South China Sea, averaged over the five years (1995, 1998, 2003, 2010, and 2013) in which the frequency of this group of clusters was most similar to that of 2020. AAF: As a fraction. Masks have been applied where the climates used in each plot were less than 1.0 mm $d^{-1}$ in (a) and (b); and 0.05 kg $m^{-1}$ $s^{-1}$ in (c–f). Green contours show the corresponding surface pressure anomaly (0.4 hPa intervals).

at the surface, appears to have been dominated by clusters characterized by anomalous high pressure over the South China Sea. The frequency of these cluster types grouped together was the greatest of all 42 years of the ERA5 dataset. Clusters characterized by low pressure in the West Pacific, which usually become more prevalent during July

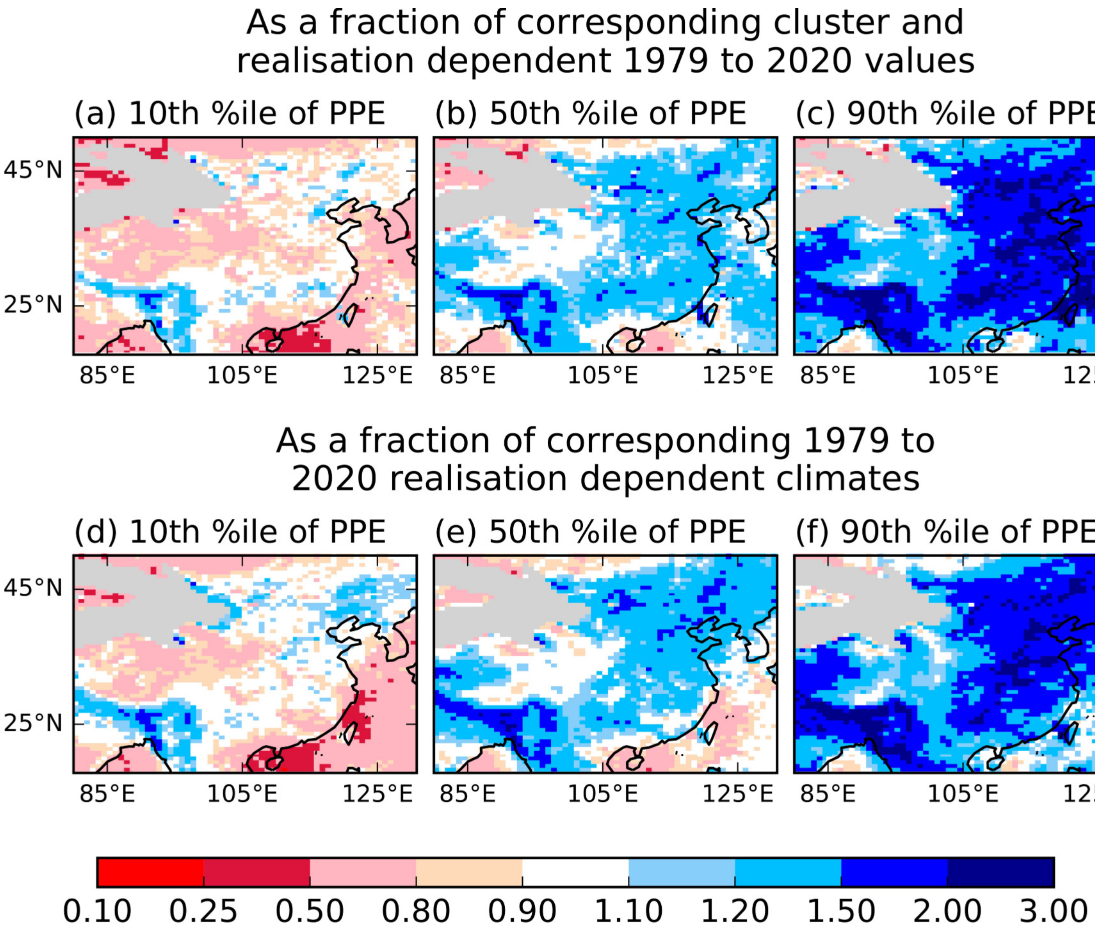

**Fig. 8**. Fractional anomalies of HadGEM3-PPE rainfall during CG1234 days of clusters of surface pressure greater than normal over the South China Sea between 15 June and 15 August for the five years of 2058 to 2099 in which these clusters were most frequent. (a) to (c) Lower, median, and upper percentile estimates (from across the PPE ensemble) as a fraction of the mean (calculated separately for each PPE simulation) of CG1234 days from the equivalent five years of 1979 to 2020 in the PPE. (d) to (f) Lower, median, and upper percentile estimates (from across the PPE ensemble) as a fraction of the mean of all days (calculated separately for each PPE simulation) of 1979 to 2020. Masks have been applied where the 1979 to 2020 values were less than 1.0 mm $d^{-1}$.

and August, were strikingly absent in 2020. This was due to the persistent westward extension of the western Pacific subtropical high.

Rainfall and water vapor flux anomalies during years of circulation cluster prevalence similar to that of 2020 were found to be remarkably similar to those of 2020, showing convincingly how the frequency of different clusters can be used as proxies for such moisture-related quantities during summer.

Simulations from two ensembles of climate models, of a plausible future world warmed by greenhouse gas concentrations, suggest that years of circulation cluster frequencies similar to 2020 are likely to become more frequent in the late 21st century. This appears to agree with Chen et al. (2020) that the western Pacific subtropical high may become stronger in the future under projected global warming. The same simulations strongly suggest that rainfall in these summers could also substantially increase. Ten percent of the simulations even suggest that increases exceeding 50% of the 2020 values are possible in the regions impacted during 2020.

***Acknowledgements***. Robin CLARK and Peili WU were supported by the UK-China Research & Innovation Partnership Fund through the Met Office Climate Science for Service Partnership (CSSP) China as part of the Newton Fund. Lixia ZHANG was supported by the National Natural Science Foundation of China under Grant No. 42075037 and the Innovative Team Project of Lanzhou Institute of Arid Meteorology (GHSCXTD-2020-2). Chaofan LI was supported by the National Key Research and Development Program of China (2018YFC1506005). We also acknowledge useful discussions with Gill MARTIN during the manuscript preparation.

***Electronic supplementary material:*** Supplementary material is available in the online version of this article at https://doi.org/10.1007/s00376-021-1086-y.

***Open Access*** This article is distributed under the terms of the Creative Commons Attribution 4.0 International License (http://creativecommons.org/licenses/by/4.0/), which permits unrestricted use, distribution, and reproduction in any medium, provided you give appropriate credit to the original author(s) and the source, provide a link to the Creative Commons license, and indicate if changes were made.

## Electronic Supplementary Material to

The Anomalous Mei-yu Rainfall of Summer 2020 from a Circulation Clustering Perspective: Current and Possible Future Prevalence

## References

[CR1] Camp J (2019). The western Pacific subtropical high and tropical cyclone landfall: Seasonal forecasts using the Met Office GloSea5 system. Quart. J. Roy. Meteor. Soc..

[CR2] Chen X L, Zhou T J, Wu P L, Guo Z, Wang M H (2020). Emergent constraints on future projections of the western North Pacific Subtropical High. Nature Communications.

[CR3] Chou L C, Chang C P, Williams R T (1990). A numerical simulation of the Mei-Yu front and the associated low level jet. Mon. Wea. Rev..

[CR4] Clark R T, Zhang L X, Li C F (2021). Clustering circulation in eastern Asia as a tool for exploring possible mechanisms of extreme events and sources of model error. Climate Dyn..

[CR5] Ding Y H, Chan J C L (2005). The East Asian summer monsoon: An overview. Meteor. Atmos. Phys..

[CR6] Eyring V, Bony S, Meehl G A, Senior C A, Stevens B, Stouffer R J, Taylor K E (2016). Overview of the Coupled Model Intercomparison Project Phase 6 (CMIP6) experimental design and organization. Geoscientific Model Development.

[CR7] Folland C K, Knight J, Linderholm H W, Fereday D, Ineson S, Hurrell J W (2009). The summer North Atlantic Oscillation: Past, present, and future. J. Climate.

[CR8] Guo Y, Wu Y, Wen B, Huang W, Ju K, Gao Y, Li S (2020). Floods in China, COVID-19, and climate change. The Lancet Planetary Health.

[CR9] Held I M, Soden B J (2006). Robust responses of the hydrological cycle to global warming. J. Climate.

[CR10] Hersbach H (2020). The ERA5 global reanalysis. Quart. J. Roy. Meteor. Soc..

[CR11] Kim B J, Kripalani R H, Oh J H, Moon S E (2002). Summer monsoon rainfall patterns over South Korea and associated circulation features. Theor. Appl. Climatol..

[CR12] Li, C., and Coauthors, 2016: Skillful seasonal prediction of Yangtze river valley summer rainfall. *Environmental Research Letters*, **11**(9), 10.1088/1748-9326/11/9/094002.

[CR13] Liu B Q, Yan Y H, Zhu C W, Ma S M, Li J Y (2020). Record-breaking Meiyu rainfall around the Yangtze River in 2020 regulated by the subseasonal phase transition of the North Atlantic Oscillation. Geophys. Res. Lett..

[CR14] Lu R Y (2001). Interannual variability of the summertime North Pacific subtropical high and its relation to atmospheric convection over the warm pool. J. Meteor. Soc. Japan. Ser. II.

[CR15] Martin G M, Dunstone N J, Scaife A A, Bett P E (2020). Predicting June mean rainfall in the middle/lower Yangtze River basin. Adv. Atmos. Sci..

[CR16] Moss, R.H., and Coauthors,2008:Towards new scenarios for analysis of emissions, climate change, impacts, and response strategies.Switzerland:IPCC.Available from http://www.ipcc.ch/pdf/supporting-material/expert-meeting-report-scenarios.pdf

[CR17] O’Neill B C (2016). The scenario model intercomparison project (ScenarioMIP) for CMIP6. Geoscientific Model Development.

[CR18] Preethi B, Mujumdar M, Kripalani R H, Prabhu A, Krishnan R (2017). Recent trends and tele-connections among South and East Asian summer monsoons in a warming environment. Climate Dyn..

[CR19] Qian W H, Lee D K (2000). Seasonal march of Asian summer monsoon. International Journal of Climatology.

[CR20] Rodwell MJ, Hoskins BJ (2001). Subtropical anticyclones and summer monsoons. Journal of Climate.

[CR21] Takaya Y, Ishikawa I, Kobayashi C, Endo H, Ose T (2020). Enhanced Meiyu-Baiu rainfall in Early Summer 2020: Aftermath of the 2019 super IOD event. Geophys. Res. Lett..

[CR22] Wang B, Fan Z (1999). Choice of South Asian summer monsoon indices. Bull. Amer. Meteor. Soc..

[CR23] Wang B, Wu Z W, Li J P, Liu J, Chang C P, Ding Y H, Wu G X (2008). How to measure the strength of the East Asian summer monsoon. J. Climate.

[CR24] Wu P L, Christidis N, Stott P (2013). Anthropogenic impact on Earth’s hydrological cycle. Nature Climate Change.

[CR25] Wu P L, Wood R, Ridley J, Lowe J (2010). Temporary acceleration of the hydrological cycle in response to a CO_2_ rampdown. Geophys. Res. Lett..

[CR26] Yamazaki K, Sexton D M H, Rostron J W, McSweeney C F, Murphy J M, Harris G R (2021). A perturbed parameter ensemble of HadGEM3-GC3.05 coupled model projections: Part 2: Global performance and future changes. Climate Dyn..

[CR27] Zhang L X, Wu P L, Zhou T J, Xiao C (2018). ENSO transition from La Niña to El Niño drives prolonged Spring-Summer drought over North China. J. Climate.

[CR28] Zheng Y G, Chen J, Ge G Q, Zhu P J (2008). Typical structure, variety, and multi-scale characteristics of Meiyu front. Acta Meteorologica Sinica.

