# Supplementary material for: The Anomalous Mei-yu Rainfall of Summer 2020 from a Circulation Clustering Perspective: Current and Possible Future Prevalence
Source: Adv Atmos Sci. 2021 Aug 31;38(12):2010–22. doi: 10.1007/s00376-021-1086-y (PMC8407136; doi:10.1007/s00376-021-1086-y)
Supplement: Supplementary file 1 — The Anomalous Mei-yu Rainfall of Summer 2020 from a Circulation Clustering Perspective: Current and Possible Future Prevalence [file 376_2021_1086_MOESM1_ESM.pdf]

Electronic Supplementary Material to: The Anomalous Mei-yu Rainfall of Summer 2020 from a Circulation Clustering Perspective: Current and Possible Future Prevalence*

Robin T. CLARK[1], Peili WU[1], Lixia ZHANG[2,3], and Chaofan LI[4]

[1]*Met Office Hadley Centre*, *FitzRoy Road*, *Exeter EX*1 3*PB*, *UK*

[2]*LASG*, *Institute of Atmospheric Physics (IAP)*, *Chinese Academy of Sciences*, *Beijing* 100029, *China*

[3]*Collaborative Innovation Center on Forecast and Evaluation of Meteorological Disasters*, *Nanjing University of Information Science & Technology*, *Nanjing* 210044, *China*

[4]*Center for Monsoon System Research*, *Institute of Atmospheric Physics*, *Chinese Academy of Sciences*, *Beijing* 10029, *China*

ESM to: Clark, R. T., P. L. Wu, L. X. Zhang, and C. F. Li, 2021: The anomalous mei-yu rainfall of summer 2020 from a circulation clustering perspective: Current and possible future prevalence. *Adv. Atmos. Sci.*, **38**(12), 2010–2022, https://doi.org/10.1007/s00376-021-1086-y.

The online version of this article can be found at https://doi.org/10.1007/s00376- 021-1086-y.

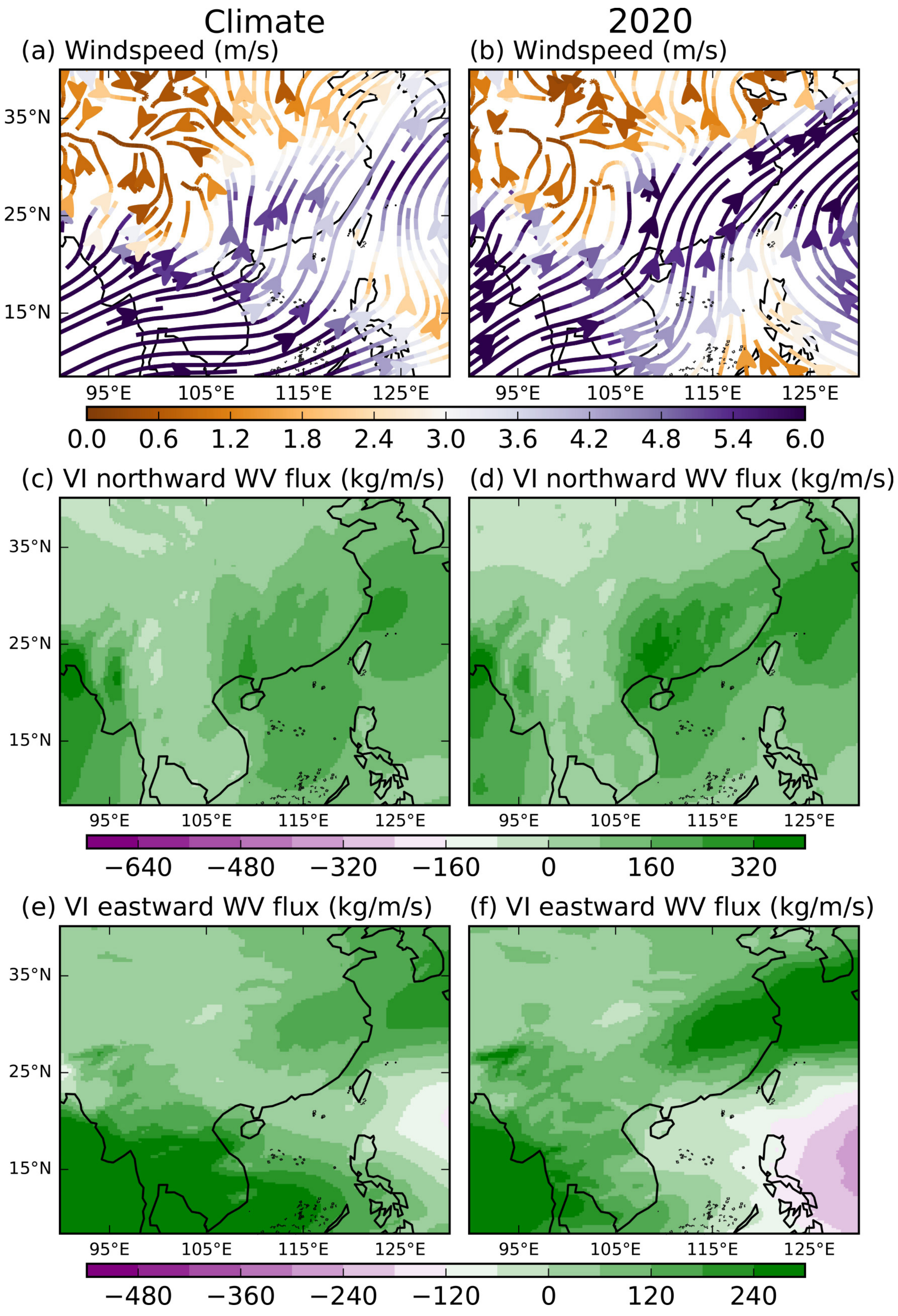

Fig. S1. (a), (c) and (e) ERA5 climates of 10 m wind speed, northward and eastward water vapor flux during the days spanning 15 June to 15 August for 1979 to 2020. (b), (d) and (f) ERA5 10 m wind speed, northward and eastward water vapor flux during 15 June to 15 August 2020.

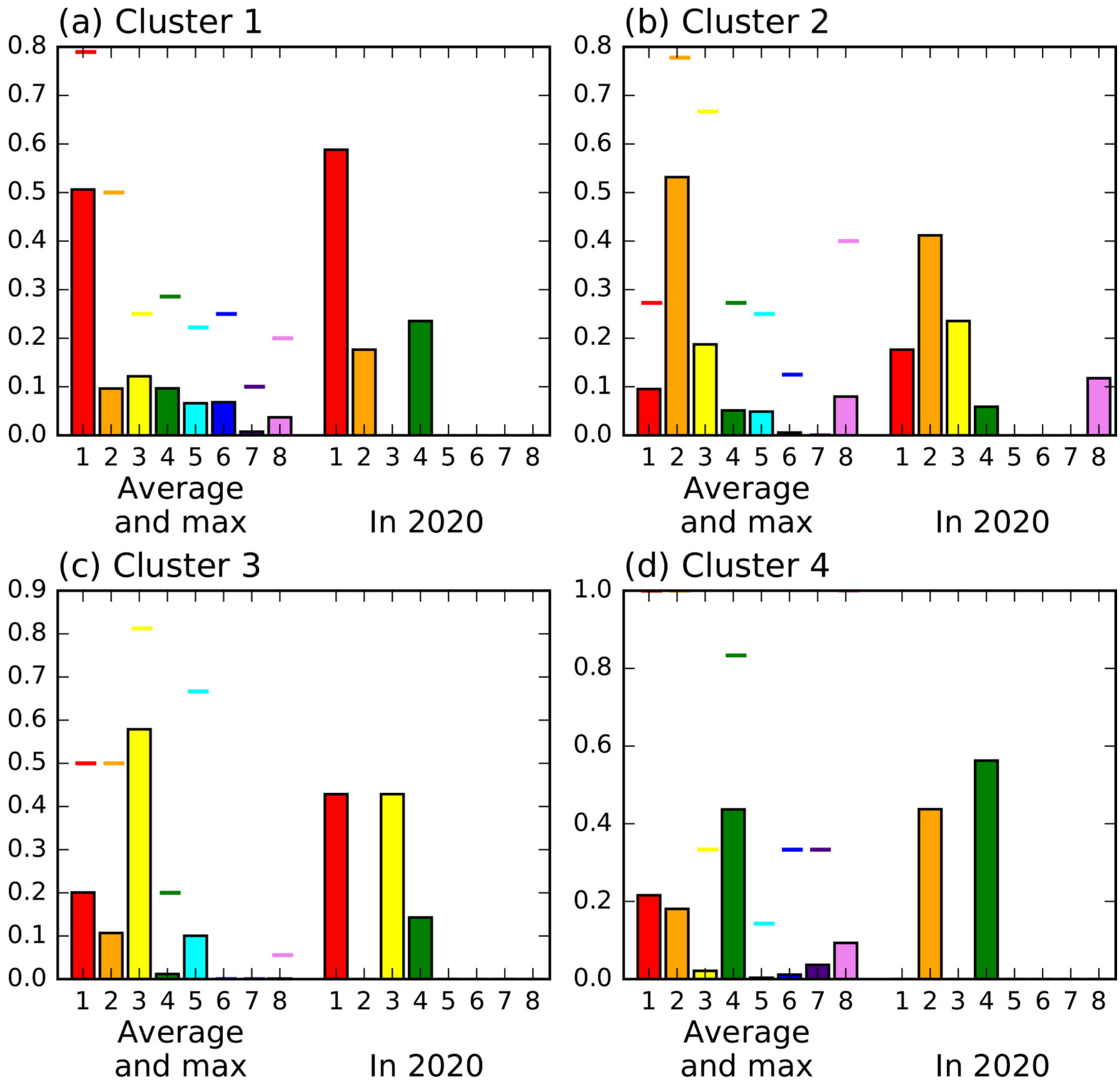

Fig. S2. Average climatological, maximum, and 2020 frequency of clusters 1 to 8 for the day immediately following 15 June to 15 August days of the most frequent clusters (clusters 1 to 4, shown in Fig. 2) of 15 June to 15 August 2020. The vertical bars show the average and 2020 values. The maximum values are shown by the horizontal colored bars. The values are given as a fraction, calculated separately for each year, of the number of days in each year following the days of the clusters 1 to 4 shown. As an example, approximately half of the days in a single year following a day of cluster 1 are climatologically also of cluster 1, while in 2020, this occurred for almost 60% of the (15 June to 15 August 2020) days of cluster 1.

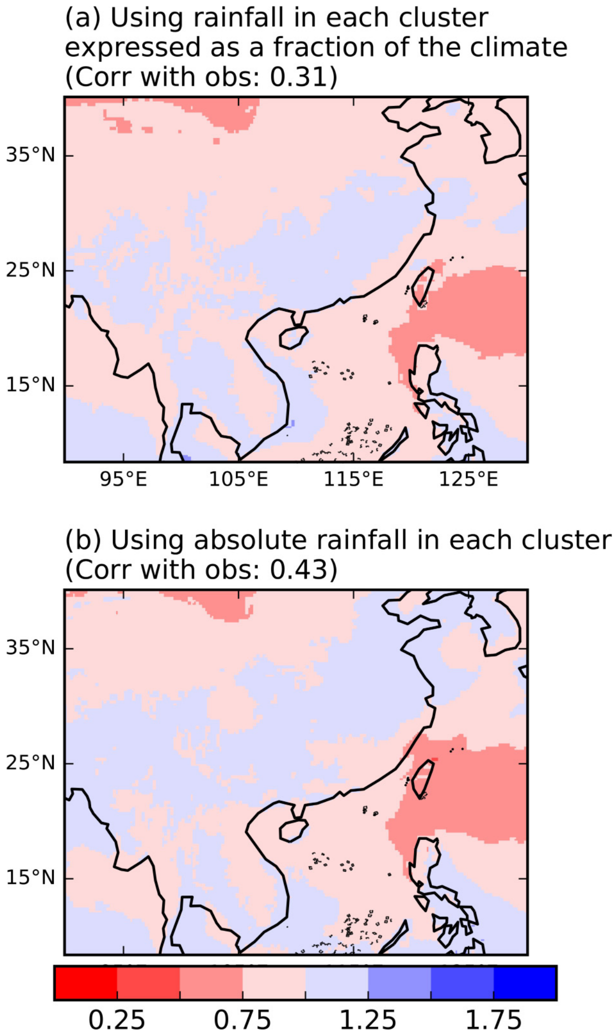

Fig. S3. Rainfall reconstructed using the rainfall in each cluster of Fig. 2 (of the main text) and the frequencies of the clusters of 15 June to 15 August 2020 shown in Fig. 3a (of the main text). The rainfall is shown as a fraction of the overall JJA climate (regardless of the cluster) using the same colors as in Fig. 1a of the main text for comparison.

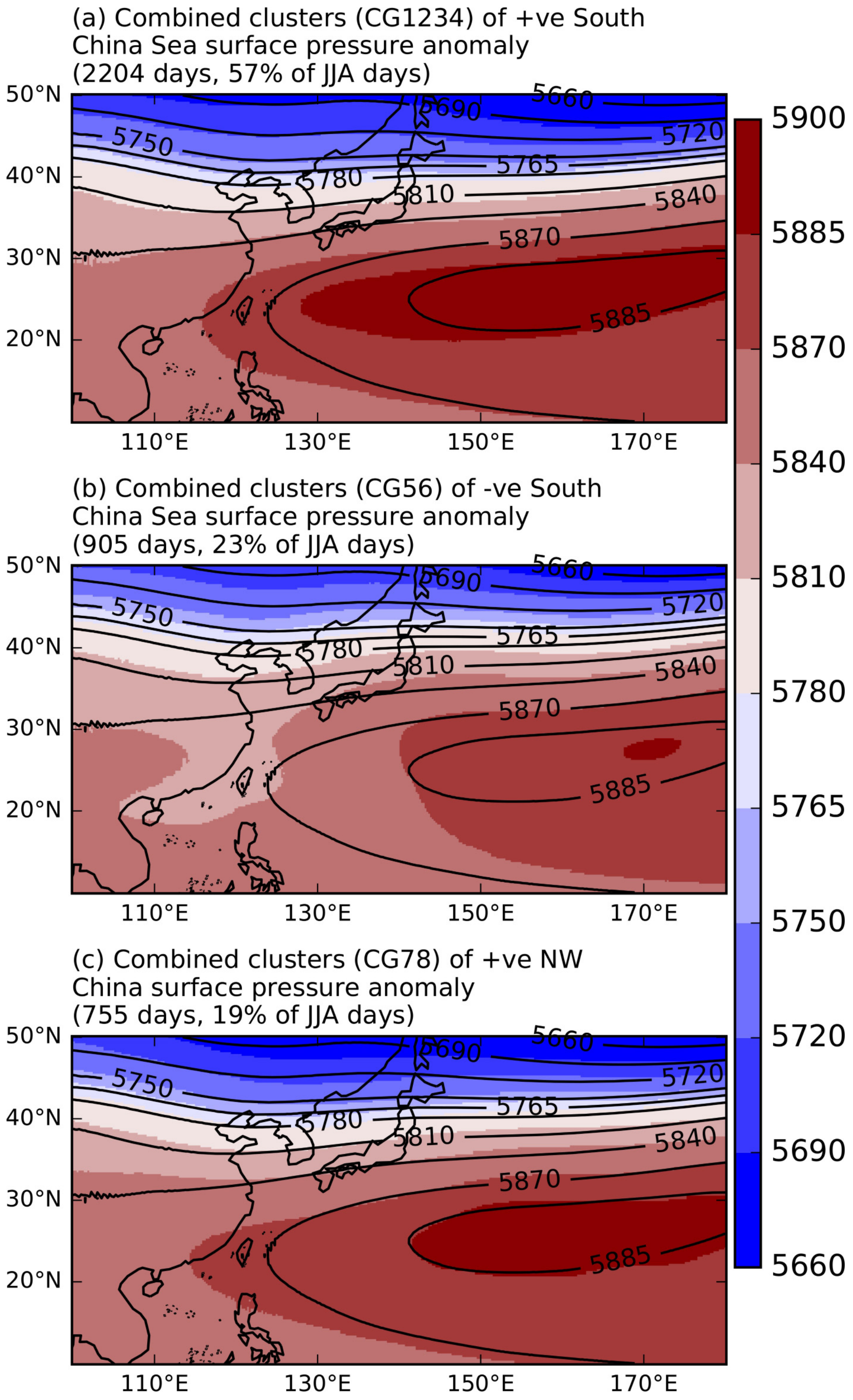

Fig. S4. Mean ERA5 500 hPa geopotential height (dam) during June, July, and August 1979 to 2020 days in (a) clusters 1, 2, 3, and 4, (b) clusters 5 and 6, and (c) clusters 7 and 8, shown in Fig. 2 (of the main text), grouped together (colored shading). Black contours give the 500 hPa geopotential height climatology. The numbers in panel titles give the overall prevalence of the cluster groups in the 3864 available JJA days of ERA5.

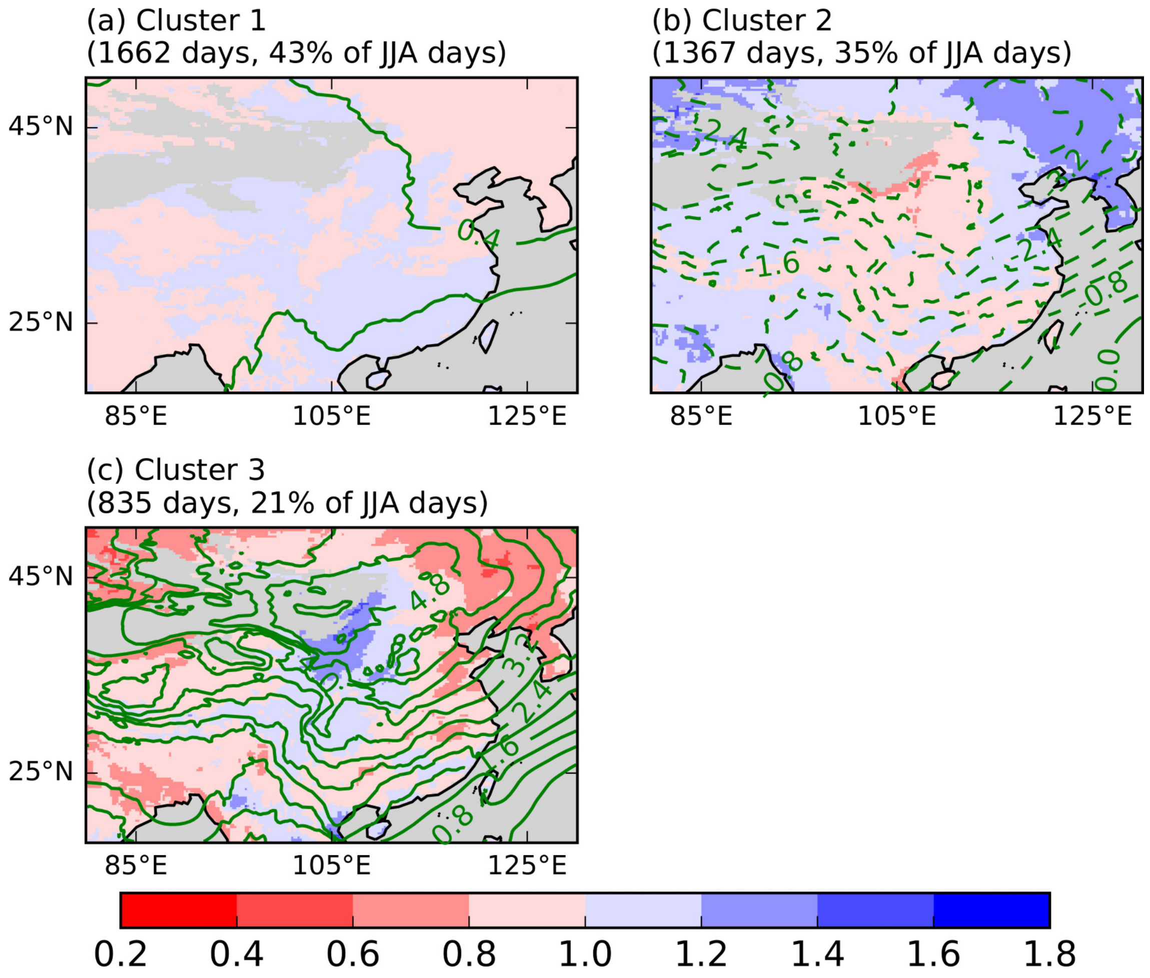

Fig. S5. ERA5 rainfall averaged over JJA 1979 to 2020 days in each cluster, as a fraction of overall (regardless of the cluster) JJA climate, when daily sea-level pressure is clustered into three clusters. A value of 2.0, for example, shows rainfall twice that of the climatology. A mask (grey) has been applied where JJA rainfall climate is less than 1.0 d^{-1}. Dashed and solid contour lines show negative and positive anomalies respectively of the mean ERA5 sea level surface pressure anomaly (0.4 hPa intervals) in each cluster.

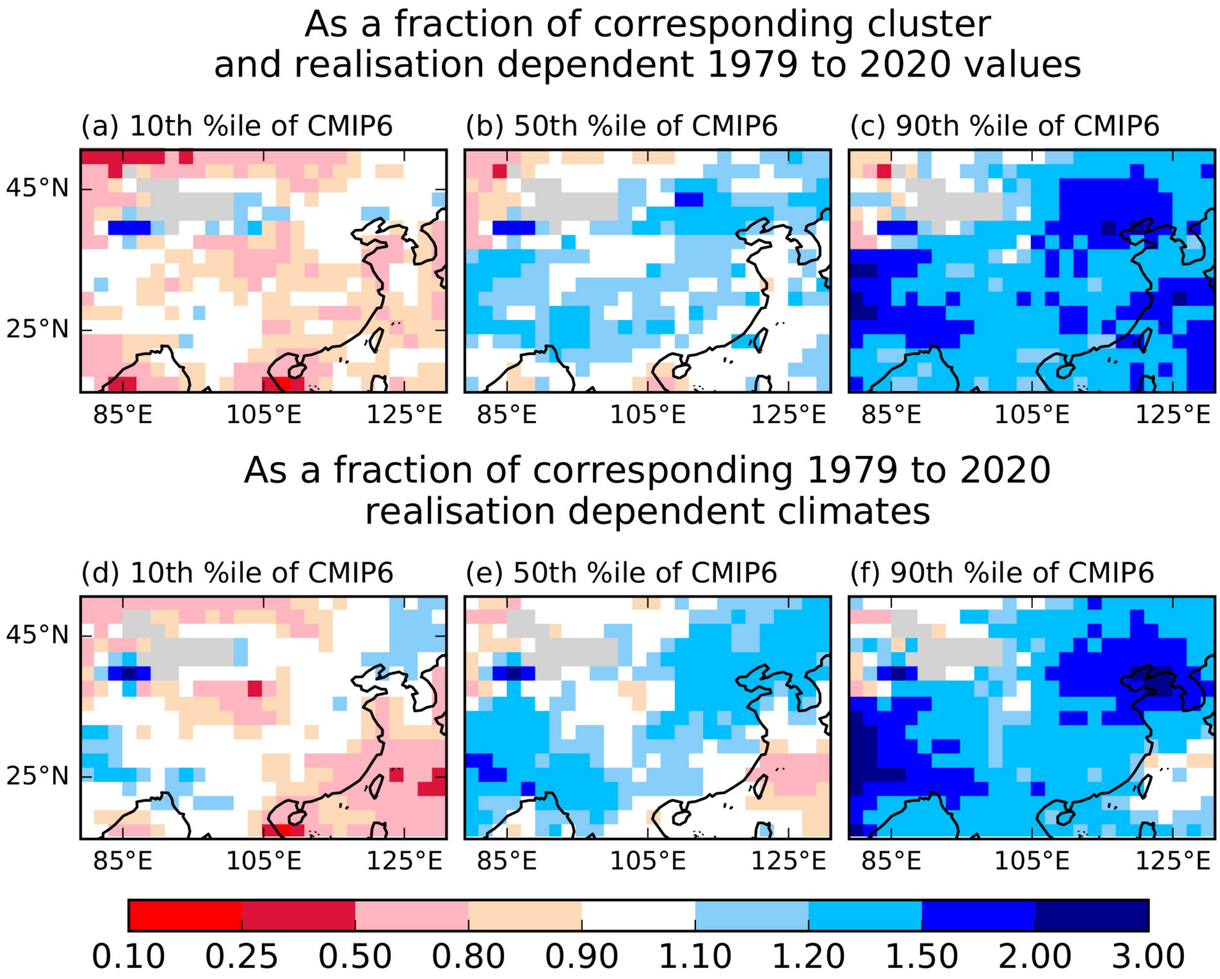

Fig. S6. Fractional anomalies of (approximately) 250 km resolution CMIP6 model rainfall during CG1234 days of clusters of surface pressure greater than normal over the South China Sea between 15 June and 15 August in the five years of 2058 to 2099 in which these clusters were most frequent. (a) to (c) lower, median and upper percentile estimates (from across the ensemble of 250 km resolution CMIP6 models) as a fraction of the mean (calculated separately for each CMIP6 realization) of CG1234 days from the equivalent five years of 1979 to 2020 in each, corresponding, CMIP6 realization. (d) to (f) lower, median, and upper percentile estimates (from across the 250 km resolution CMIP6 ensemble) as a fraction of the mean of all days (calculated separately for each CMIP6 simulation) of 1979 to 2020. A mask (grey) has been applied where the 1979 to 2020 values are less than 1.0 mm d^{-1}.
